# Rapid Increase of Scrub Typhus, South Korea, 2001–2006

**DOI:** 10.3201/eid1507.080399

**Published:** 2009-07

**Authors:** Sun-Seog Kweon, Jin-Su Choi, Hyun-Sul Lim, Jang-Rak Kim, Keon-Yeop Kim, So-Yeon Ryu, Hyo-Soon Yoo, Ok Park

**Affiliations:** Chonnam National University Hwasun Hospital, Hwasun, South Korea (S.-S. Kweon); Chonnam National University College of Medicine, Gwangju, South Korea (J.-S. Choi); Dongguk University College of Medicine, Gyeongju, South Korea (H.-S. Lim); Gyeongsang National University School of Medicine, Jinju, South Korea (J.-R. Kim); Kyungpook National University School of Medicine, Daegu, South Korea (K.-Y. Kim); Chosun University College of Medicine, Gwangju (S.-Y. Ryu); Korea Centers for Disease Control and Prevention, Seoul, South Korea (H.-S. Yoo, O. Park)

**Keywords:** Rickettsia, scrub typhus, vector-borne infections, South Korea, tsutsugamushi disease, letter

**To the Editor:** Scrub typhus, or tsutsugamushi disease, is a febrile illness caused by the rickettsial bacteria *Orientia tsutsugamushi*. Scrub typhus is endemic to a geographically distinct region, the so-called tsutsugamushi triangle, which includes Japan, Taiwan, China, and South Korea (*1*,*2*). Scrub typhus is a public health issue in Asia, where 1 billion persons may be at risk for the disease (*3*). In South Korea, scrub typhus is the most common rickettsial disease, and public health authorities are concerned about its increased incidence.

Scrub typhus has been a reportable disease in South Korea since 1994. Physicians who diagnose suspected or confirmed cases must report these cases to their local health bureau and the Korea Centers for Disease Control and Prevention (KCDC) through the National Notifiable Disease Surveillance System (NNDSS). For a patient’s illness to meet the case definition for scrub typhus, the clinical signs (acute febrile illness and skin eschar) must be present or there must be laboratory confirmation (4-fold rise in antibody titer, antigen detected in blood, or genetic material detected by PCR).

We analyzed NNDSS data confirmed by KCDC and classified all reported cases into 2 groups according to residential area. Cases with rural administrative address codes “eup” or “myun” were defined as rural cases, whereas cases with a city administrative address code of “dong” were defined as urban cases. All case-patients were classified by occupation as farmer or nonfarmer; all agricultural, fishery, and forest workers from rural areas were defined as farmers.

In total, 23,929 cases, including 16,199 (67.7%) serologically confirmed cases, were reported between 2001 and 2006, of which 35.5% were male patients and 64.5% female patients. The greatest number of cases was in the age group 50–69 years, in both male (47.2%) and female (51.7%) patients; however, there were 167 boys (2.0%) and 119 girls (0.8%) <10 years of age. The number of cases peaked in 2005, with 2,331 and 4,449 cases in male and female patients, respectively. In 2006, a total of 6,480 cases (2,364 and 4,116 in males and females patients), which is 2.5× the number reported in 2001, were reported. The autumn epidemic period was from October through November; 96.2% of all cases were reported during this period (Figure). The proportion of cases identified in farmers decreased from 2001 (44.4%) to 2006 (36.4%); the number of cases in nonfarmers reached 4,121 (63.6%) in 2006. The number and proportion of patients living in urban areas increased from 1,059 (40.2%) in 2001 to 3,230 (49.9%) in 2006. This trend was observed in both farmers and nonfarmers. The number of cases among farmers living in urban areas increased from 150 (12.8%) to 443 (18.8%), while the corresponding number of cases in nonfarmers went from 909 (62.0%) to 2,787 (67.6%). In addition, we identified different features of scrub typhus epidemicity, compared with those reported in previous studies (*4*–*7*). Many of the values reported in this study (64.5% of cases in female patients, 59.5% in nonfarmers, and 96.2% occurring in autumn) are higher than the values reported previously in Japan (*4*), Taiwan (*5*), and China (*6*). The higher incidence in female workers may be associated with conventional South Korean working behavior. Female workers typically work in a squatting position, with bare hands, and usually in dry fields, whereas male workers tend to work in a standing position, with tools, and in rice fields. Therefore, female workers are more likely to be exposed to infected mites.

**Figure:**
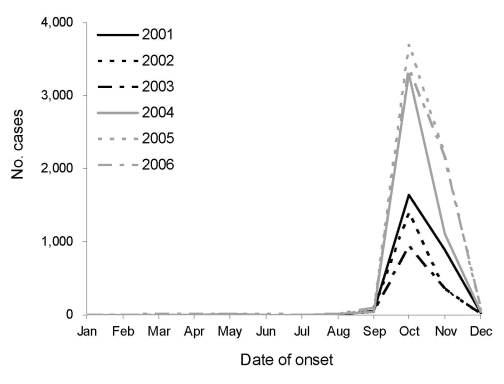
Monthly occurrence of scrub typhus cases in South Korea, 2001–2006.

Previously, farmers were considered a high-risk group, but our results imply that the same or even more attention should be given to nonfarmers. *Leptotrombidium pallidum*, a common mite in Korea, first appears in September. Its population then peaks in October and November and to a lesser degree in April and May (*7*). In autumn, especially around Chusok (Korean Thanksgiving), nonfarmers and urban residents also take part in agricultural activities, such as the chestnut harvest, mowing around graves, and assisting their farmer relatives. A sharp peak in the number of cases occurred during October–November, which is inconsistent with a previous report on vector density showing a secondary peak during April–May (*7*). This finding suggests that many cases are misreported, especially in spring. Unfortunately, there are still no reports on the comprehensiveness of the scrub typhus surveillance system in South Korea. We cannot exclude other modes of exposure such as golf, climbing, and other outdoor leisure activities. A 5-day work week was introduced in 2004, and, as a result, more leisure time has been available to urban residents. In addition, improved surveillance and diagnostic methods as well as changes in atmospheric temperature (*8*) may have contributed to the increase.

We report the rapid increase of scrub typhus and the proportion of infected persons living in urban areas in South Korea. This information will be used to establish strategies for prevention, surveillance, and management in South Korea and in other countries where scrub typhus is endemic.
